# Molecular Characterization of German *Acinetobacter baumannii* Isolates and Multilocus Sequence Typing (MLST) Analysis Based on WGS Reveals Novel STs

**DOI:** 10.3390/pathogens10060690

**Published:** 2021-06-01

**Authors:** Gamal Wareth, Jörg Linde, Philipp Hammer, Wolf D. Splettstoesser, Mathias W. Pletz, Heinrich Neubauer, Lisa D. Sprague

**Affiliations:** 1 Friedrich-Loeffler-Institute, Institute of Bacterial Infections and Zoonoses (IBIZ), Naumburger Str. 96a, D-07743 Jena, Germany; joerg.linde@fli.de (J.L.); heinrich.neubauer@fli.de (H.N.); lisa.sprague@fli.de (L.D.S.); 2 Institute for Infectious Diseases and Infection Control, Jena University Hospital, Am Klinikum 1, D-07747 Jena, Germany; Mathias.Pletz@med.uni-jena.de; 3 Bacteriology, Immunology, and Mycology Department, Faculty of Veterinary Medicine, Benha University, Moshtohor, Toukh 13736, Egypt; 4 Department of Microbiology and Biotechnology, Max Rubner-Institut, Hermann-Weigmann-Str. 1, D-24103 Kiel, Germany; Philipp.Hammer@mri.bund.de; 5 Department of Microbiology & Hygiene, LADR GmbH, Medical Laboratory Braunschweig, 38124 Braunschweig, Germany; w.splettstoesser@LADR.de; 6 Research Campus Infectognostics, Philosophenweg 7, 07743 Jena, Germany

**Keywords:** *Acinetobacter baumannii*, identification, MLST, WGS, sequence types, Germany

## Abstract

*Acinetobacter baumannii* (*A.* *baumannii*) is a major cause of severe nosocomial infections worldwide. The emergence of infections associated with *A.* *baumannii* poses a significant health risk in Germany. *A. baumannii* is part of the ACB complex and is difficult to distinguish from other species phenotypically, necessitating its reliable identification. The current study analyzed 89 *A. baumannii* strains from human and non-human origins by matrix-assisted laser desorption/ionization (MALDI–TOF) and PCR detection of intrinsic *bla*_OXA-51-like_ carbapenemase, *bla*_OXA-23-like_, *bla*_OXA-24-like_, *bla*_OXA-58-like_, and IS*Aba* 1 genes. Whole-genome sequencing (WGS) was applied for species confirmation and strain type determination. Combining the molecular detection of the intrinsic *bla*_OXA-51-like_ carbapenemase gene together with MALDI–TOF with a score value of >2.300 proved to be a suitable tool for *A. baumannii* identification. WGS data for all of the sequenced strains confirmed the identity of all *A. baumannii* strains. The Pasteur scheme successfully assigned 79.7% of the strains into distinct STs, while the Oxford scheme succeeded in allocating only 42.7% of isolates. Multilocus sequence typing (MLST) analysis based on the Pasteur scheme identified 16 STs. ST/241 was the most prevalent in samples from non-human origin, whereas ST/2 was predominant in human samples. Furthermore, eight isolates of non-human origin were allocated to seven new STs (ST/1410, ST/1414, ST/1416, ST/1417, ST/1418, ST/1419, and ST/1421). Ten isolates from non-human origin could not be typed since new alleles were observed in the loci Pas_*cpn*60, Pas_*rpo*B, and Pas_*glt*A. MLST analysis based on the Pasteur scheme was more appropriate than the Oxford scheme for the current group of *A. baumannii.*

## 1. Introduction

*Acinetobacter baumannii *(*A. baumannii*) is a Gram-negative, pleomorphic, and non-motile opportunistic bacterial pathogen. Multi-drug resistant (MDR) *A. baumannii* strains are the cause of both hospital- and community-acquired infections worldwide [1]. In Germany, several outbreaks have occurred among hospitalized patients [2] and in veterinary clinics [3,4,5], and a recent study identified *A. baumannii* in dried milk [6,7]. The impact of *A. baumannii* in veterinary medicine and food chains is not well understood [8]. The definitive identification of *A. baumannii* is challenging. *A. baumannii* is part of the *A. calcoaceticus–A. baumannii* complex (ACB complex) whose members are difficult to distinguish phenotypically. This complex encompasses six species: *A. calcoaceticus*, *A. baumannii*, *A. pittii*, *A. nosocomialis*, and the recently included species *A. seifertii* and *A. dijkshoorniae* [9,10]. Phenotypic tests such as VITEK 2 and API 20NE are of limited use and fail to distinguish between different members of the *A. baumannii* complex [11]. Given the clinical significance of *A. baumannii*, accurate species identification and correct differentiation from other ACB complex members, particularly the closely related species *A. pittii* and *A. nosocomialis,* is essential.

The approach known as average nucleotide identity (ANI) for identification, together with multilocus phylogenetic analysis based on Next Generation Sequencing (NGS) technology, currently seems to be the best alternative for a gold standard and the most promising method [12]. In addition, the Kraken program, which works with WGS data, is considered a highly accurate and ultrafast tool for taxonomic classification [13]. Multilocus sequence typing (MLST) has been used for the molecular typing of bacterial isolates. The population structure of *A. baumannii* strains has been studied using two known seven-gene MLST schemes, the Oxford scheme [14] and the Pasteur scheme [15]. Both are based on fragments of seven housekeeping genes. In spite of the fact that the two schemes share three genes, they form two coexisting nomenclatures of sequence types and clonal complexes [16]. Previous work recommended the Oxford scheme due to the presence of the *gpi* gene, which can provide a link between typing and phenotypic information [17]. However, having all seven of the genes in the Oxford scheme in one half of the chromosomes [18] makes the Pasteur scheme more appropriate for epidemiological studies of *A. baumannii* [16].

The present study analyzed 89 *A. baumannii* strains from human and non-human origins using a combination of MALDI–TOF, different PCR protocols, detection of the intrinsic *bla*_OXA-51-like_ carbapenemase gene, whole-genome sequencing (WGS), and multilocus sequence typing (MLST).

## 2. Materials and Methods

### 2.1. Bacterial Isolates

Eighty-nine unique, non-repetitive *A. baumannii* strains were obtained from the strain collection of the Institute of Bacterial Infections and Zoonoses (IBIZ), Jena. Each isolate was obtained from a unique sample. Fourteen were clinical isolates from human origin, and two were strains of animal origin belonging to the German resistance monitoring system of animal-pathogenic bacteria (GERM-Vet-2016). Seventy-one isolates were isolated at the production level from the end product of powdered milk samples produced by three different companies in Germany, and two *A. baumannii* control strains (DSM30007 and DSM105126) from human origin were included. The strains were isolated between 2005 and 2018 [7].

### 2.2. Identification and Molecular Characterization of Strains

The microbial species identification was made using Matrix-Assisted Laser Desorption/Ionization Mass Spectrometry (MALDI–TOF MS). Briefly, each isolate was cultured on Columbia blood agar at 37 °C, and a single fresh colony from each pure culture plate was suspended in a 1.5 mL Eppendorf tube containing 300 µL of HPLC grade water. The bacteria were inactivated by 900 µL of absolute ethanol. Protein extraction from each sample and measurement was performed as described before [18]. The following MALDI log score values for species identification were applied: score values between 0 and 1.690 were considered ‘no reliable identification’; values between 1.700 and 1.990 were considered ’probable genus identification’; values between 2.000 and 2.290 were considered a ’secure genus identification and probable species identification’; and values equal to or greater than 2.300 were considered highly probable for species identification. Since the intrinsic *bla*_OXA-51-like_ carbapenemase genes are present in the vast majority of *A. baumannii* strains, the *bla*_OXA-51-like_ PCR using the protocol and primers established by Turton et al., 2006 [19], was applied to confirm the species identification of all positive MALDI strains. Moreover, three subgroups of acquired OXA carbapenemases (*bla*_OXA-23-like_, *bla*_OXA-24-like_, and *bla*_OXA-58-like_) were used in the protocol established by Woodford et al., 2006 [20], and multiplex PCR was used for the *bla*_OXA_ and IS*Aba* 1 genes prevalent in *Acinetobacter* spp. using the protocol of Kobs et al., 2016 [21]. The genotyping and characterization of all bacterial isolates were performed by average nucleotide identity (ANI) [12] and the Kraken program [13] utilizing the whole-genome-sequencing (WGS) data.

### 2.3. DNA Extraction and Whole-Genome Sequencing (WGS)

Genomic DNA was extracted from a single colony grown on Columbia blood agar at 37 °C using the High Pure PCR Template Preparation Kit (Roche Diagnostics GmbH, Mannheim, Germany) according to the manufacturer’s instructions. The sequencing library was prepared, and the library was sequenced using a MiSeq sequencer (Illumina, San Diego, CA, USA). Analysis of the raw data, quality control, and assembly were performed utilizing the Linux-based pipeline WGSBAC (v2.0.0) (https://gitlab.com/FLI_Bioinfo/WGSBAC (accessed on 1 January 2020)), as previously described [7]. WGSBAC utilizes Kraken (v. (2.0.7_beta) [22] to classify reads at the genus and species levels and to calculate coverage, the number of reads multiplied with their average read length and divided by the genome size. WGSBAC performs genome assembly with Shovill (v. 1.0.4) that is based on the SPAdesassembler [23] and determines the quality of assemblies with QUAST (v. 5.0.2) [24].

### 2.4. Multilocus Sequence Typing (MLST)

As a general overview for genotyping, WGSBAC performs classical Multilocus Sequence Typing (MLST, https://github.com/tseemann/mlst, v. 2.16.1, accessed on 1 January 2020) on assembled *A. baumannii* genomes utilizing the two widely used and available MLST schemes. The first scheme (*Acinetobacter baumannii*#1) is referred to as the Oxford scheme and was developed and evaluated by Bartual and coworkers [14]. This scheme encompasses 2078 distinct sequence types (ST1-ST2078, Last updated 1 January 2020). It is designed to identify the following seven internal house-keeping genes: citrate synthase (*gltA*), DNA gyrase subunit B (*gyrB*), glucose dehydrogenase B (*gdhB*), homologous recombination factor (*recA*), 60-kDa chaperonin (*cpn60*), glucose-6-phosphate isomerase (*gpi*), and RNA polymerase sigma factor (*rpoD*). In comparison, the second scheme (*Acinetobacter baumannii*#2) was published later by Diancourt and coworkers and is referred to as the Pasteur scheme [15]. This scheme encompasses 1409 distinct sequence types (ST1-ST1409, last updated accessed on 1 January 2020) and uses fragments of seven internal housekeeping genes. Three genes are shared with the Oxford scheme (*cpn60, gltA*, and *recA*), and the four other unique genes are elongation factor EF-G (*fusA*), CTP synthase (*pyrG*), 50S ribosomal protein L2 (*rplB*), and RNA polymerase subunit B (*rpoB*). To determine and fully describe the novel alleles found with less than 100% identity, the same assembled genomes of all new STs were typed using another MLST web-server (https://cge.cbs.dtu.dk/services/MLST/, 1 January 2020) belonging to the Center for Genomic Epidemiology. The allelic variants of all gene fragments of both schemes were determined from the tested strains’ genomes, and the contig files were scanned against traditional PubMLST typing schemes. The alignment detected the mismatches nucleotides and the nearest STs against the Pasteur MLST scheme’s allele with the best alignment score.

## 3. Results

### 3.1. Identification of A. baumannii Strains

All examined strains in the current study were initially identified as *A. baumannii* with a score value of >2.300 by MALDI–TOF analysis. PCR confirmed the presence of the *bla*_OXA-51 like_ carbapenemase gene in all isolates from human, animal, and milk powder samples. None of *bla*_OXA-23-like_, *bla*_OXA-24-like_, *bla*_OXA-58-like_, and IS*Aba*1 was confirmed by PCR in strains obtained from milk samples. In contrast, IS*Aba*1 and *bla*_OXA-23-like_ were found in almost all of the tested isolates of human origin. Whole-genome sequencing additionally confirmed correct strain identification. Average nucleotide identity (ANI) was calculated for all sequenced strains and confirmed the identity of *A. baumannii* with values more than the cut-off values (95–96%) recommended by Richter and Rossello-Mora [25]. Using the Kraken program for genus identification, the first match for all isolates was always “*Acinetobacter*” for an average of 97.87% of the reads, and for species level, the first match for all isolates was always “*Acinetobacter baumannii*”.

### 3.2. MLST Analyses

MLST analyses based on the Pasteur scheme identified 16 sequence types (ST) for 71/89 strains (79.7%) (Table 1). Of the eighteen strains (20.3%), which initially could not be assigned to a distinct sequence type, eight were allocated to seven new STs (Table 2). Ten isolates originating from milk powder samples could not be typed due to new alleles. These new alleles mostly occurred in the loci Pas_*cpn*60, Pas_*rpo*B, and Pas_*glt*A (Table 3). In the 71 isolates derived from milk powder samples, ST/241 was the most prevalent sequence type, followed by ST/153, ST/40, ST/1119, and ST/273. ST/33 and ST/364 were determined once. Among the fourteen isolates of human origin, eight (9%) were assigned to ST/2; two were assigned to ST/164; and one each was assigned to ST/1, 78, 215, and 604. The control strains DSM30007 and DSM105126 from human origin were assigned to ST/52 and ST/437. One strain of animal origin was assigned to ST/46, and the other, which resulted as an unknown ST, was later assigned to the new ST/1410.

Using the Oxford scheme, thirty-eight strains (42.7%) were assigned to ten STs, while fifty-one strains (57.3%) could not be assigned to a distinct sequence type and were thus defined as unknown STs (Table 4). ST/613 was the most prevalent sequence type in the isolates originating from milk powder samples, followed by ST/427. One isolate was assigned to ST/1182. Among the fourteen isolates of human origin, two (2.3%) were assigned to ST/1418 and one each was assigned to ST/195, 348, 806 and ST/944. Eight strains could not be assigned to a sequence type and were defined as unknown STs. The two control strains DSM30007 and DSM105126, of human origin, were assigned to ST/112 and ST/931. Two strains of ST/2 in the Pasteur scheme were assigned to two different STs in the Oxford scheme, i.e., ST/2 resulted as ST/195 and ST/348, respectively. The seven new STs determined using the Pasteur scheme (ST/1410, 1414, 1416-1419, and ST/1421) could not be typed using the Oxford scheme except for one, which was assigned as ST/1182. The new alleles occurred mainly at the loci Oxf_*cpn*60, Oxf_*gdh*B, Oxf_*gpi*, and Oxf_*rpo*D (Table 5).

## 4. Discussion

*Acinetobacter baumannii* is a serious and emerging pathogen, causing nosocomial infections in humans and animals in Germany [2,5,7,8]. Accurate identification and differentiation of *Acinetobacter* at the species level is challenging. Identification of *A. baumannii* is mainly based on the semi-automated Vitek-2 system and MALDI–TOF. The failure of the Vitek-2 system to correctly identify *A. baumannii* is well known [11]. MALDI–TOF log score values of >2.000 have been accepted in some laboratories for species identification [26]. However, only score values between 2.3 and 3.0 indicate ‘highly probable species identification’, and values between 2.0 and 2.29 indicate a ‘secure genus identification and probable species identification’ [18]. The phenotypic techniques using standard biochemical methods and automated systems currently available are insufficient [27], leading to inaccurate identification. Different PCR protocols were used to identify subgroups of acquired and intrinsic OXA carbapenemase genes in the current study. A combination of molecular characterization using the *bla*_OXA-51-like_ carbapenemase gene PCR, according to Turton et al. [19], together with MALDI–TOF with score values of 2.300 or more provided a highly probable and suitable tool for *A. baumannii* identification. The obtained results are in agreement with those described by Szabados and coworkers, who demonstrated that the species-specific score cut-off values >2.300 of clinical *A. baumannii* represent a secure score value for species identification [28]. The present study confirmed the presence of the *bla*_OXA-51-like_ carbapenemase gene in all *A. baumannii* isolates and the usefulness of this PCR assay [29]. The species misidentification may negatively influence clinical outcomes. A proper and accurate molecular identification of *A. baumannii* into species level is needed, and typing methods with high discriminatory power are crucial for diagnosis [11,30].

Conventional MLST and PFGE are effective methods to describe bacterial populations and are considered the gold standard of typing. However, they are time consuming and expensive. In the current study, we replaced the conventional MLST methodology with digital MLST based on WGS data. Traditional MLST was designed to identify the sequence types, but it provided no in-depth information regarding the novel alleles found, percentage of matching, or locus allele alignment details with the changes in nucleotides that can be obtained from MLST based on WGS data. Multilocus sequence typing analysis confirmed the considerable diversity of *A. baumannii* in the investigated strains. In the current study, the Pasteur scheme successfully assigned 71 (79.7%) of the strains into distinct STs while the Oxford scheme succeeded in allocating only 38 (42.7%) of isolates. The results in the present study are in agreement with the recent observations of Gaiarsa et al., who demonstrated that the Pasteur scheme is more appropriate for population biology, precise strain classification, and epidemiological studies of *A. baumannii* [16]. They also proposed it to be the scheme of choice in parallel with the core genome MLST (cgMLST) schemes that consist of a fixed set of conserved genome-wide genes and are usually species-specific when used for very closely related bacterial species.

The published knowledge on the general distribution of STs of *A. baumannii* in the German population is scarce. Few STs of *A. baumannii* from human origin have been published. The MLST analyses showed that more than half of human strains (eight out of fourteen) in the current study belonged to ST/2 (Pasteur). ST2 belongs to international clone II and is the most dominant type globally [31]. ST2 was the only ST identified in an outbreak involving ten patients in Leverkusen [32]. Together with ST/113 and ST/325 (Pasteur), it was reported in strains collected between 2012 and 2015 in patients at Heidelberg University Hospital [33]. Moreover, the ST2/ST348 and ST78/ST944 strains (Pasteur/Oxford), respectively, were determined from two patients hospitalized in Bavaria and Hesse [34] and were determined in two strains in the current study. The existence of ST/2 (Pasteur) is not limited to humans, and it was the dominant ST in strains isolated from pet animals in Germany [3] and from sheep in Pakistan in 2012 [35]. One strain from human origin was assigned to ST/1 (Pasteur). ST/1 was identified in a strain obtained from a cat in Germany in 2000 [36]. ST/195 (Oxford) was identified in a strain of human origin. ST/195 was reported together with ST/218 (Oxford) in an outbreak involving ten patients in Leverkusen [32]. Additionally, ST22 and ST53 (Oxford scheme) were identified in an outbreak encompassing seven patients in Rostock [37].

In samples originating from milk powder, ST/241, ST/153, and ST/40 (Pasteur) were the predominant STs. ST/241 and ST/40 were identified recently in *A. baumannii* isolates obtained from bovine in Hesse [26]. However, both STs have not yet been found in human samples in Germany. ST427 and ST/613 (Oxford) were the predominant STs. Both STs were identified in samples obtained from powdered milk produced in Germany [6]. However, they are uncommon in human strains in Germany. On the other hand, ST/836 (Oxford) and ST/388 (Pasteur) have been detected in two isolates recovered from sewage water from a poultry slaughterhouse in Germany [38]. None of the identified STs was shared between the human and non-human strains.

## 5. Conclusions

To summarize, we recommend utilizing the MALD-TOF with a score value >2.300 and *bla*_OXA-51-like_ PCR together with molecular identification based on WGS data for accurate identification, and differentiation of *A. baumannii.* WGS assembly-based approaches have complementary characteristics over conventional methods. The detection and characterization of ST must move from PCR to high throughput identification via sequencing and in silico detection using freeware programs and public databases. Eleven new STs (7 novel and 4 untyped) were identified in German *A. baumannii* isolates. The Pasteur scheme is the more appropriate scheme than the Oxford scheme, at least in the current tested group of *A. baumannii*.

## Figures and Tables

**Table 1:** Strain types (ST) of 89 *A. baumannii* strains isolated in Germany according to the Pasteur scheme.

Pasteur ST.	No. of Isolates	%	Origin of Isolates
ST/241	19	21.4	Milk powder sample
ST/153	12	13.5	Milk powder sample
ST/40	12	13.5	Milk powder sample
ST/1119	6	6.7	Milk powder sample
ST/273	3	3.4	Milk powder sample
ST/364	1	1.1	Milk powder sample
ST/33	1	1.1	Milk powder sample
ST/2	8	9	Human
ST/164	2	2.3	Human
ST/604	1	1.1	Human
ST/215	1	1.1	Human
ST/78	1	1.1	Human
ST/1	1	1.1	Human
ST/52	1	1.1	DSM30007 (Human)
ST/437	1	1.1	DSM105126 (Human)
ST/46	1	1.1	Animal
ND	18	20.3	17 Milk powder sample/1 Animal
Total	89	100	

ST, sequence type; D, not determined.

**Table 2:** Description of the new STs found in the current study based on Pasteur scheme (*Acinetobacter baumannii*#2).

STs	ID	Origin	Year	*cpn*60	*fus*A	*glt*A	*pyr*G	*rec*A	*rpl*B	*rpo*B
**1410**	18Y0036	Animal	2016	40	3	2	1	2	2	2
**1414**	18Y0093	Milk powder	2005	3	3	6	2	3	1	2
	18Y0112	Milk powder	2005	3	3	6	2	3	1	2
**1416**	18Y0111	Milk powder	2005	3	3	2	2	2	4	14
**1417**	18Y0141	Milk powder	2006	3	3	2	2	9	2	2
**1418**	18Y0169	Milk powder	2007	3	2	2	2	3	1	2
**1419**	18Y0192	Milk powder	2009	156	3	56	1	13	1	1
**1421**	18Y0245	Milk powder	2012	69	4	2	2	7	1	4

**Table 3:** Allelic profiles of the untyped strains found in the current study based on the Pasteur scheme (*Acinetobacter baumannii*#2).

	ID	Origin	Year	*cpn*60	*fus*A	*glt*A	*pyr*G	*rec*A	*rpl*B	*rpo*B	Novel Allele *
**UT**	18Y0092	Milk powder	2005	1	2	**~140**	1	51	1	4	(Pas_*glt*A_2 G >A)
**UT**	18Y0103	Milk powder	2005	**~155**	3	13	1	3	1	**~14**	(Pas_*cpn*60_25 G >A) (Pas_*rpo*B_14 C >T)
	18Y0107	Milk powder	2005	**~155**	3	13	1	3	1	**~14**	
	18Y0237	Milk powder	2011	**~155**	3	13	1	3	1	**~14**	(Pas_*cpn*60_46 A>G) (Pas_*rpo*B_14 C>T)
	18Y0228	Milk powder	2011	**~155**	3	13	1	3	1	**~14**	(Pas_*cpn*60_155C>T) (Pas_*rpo*B_14 C>T)
**UT**	18Y0195	Milk powder	2009	3	2	2	**~2**	5	1	5	(Pas_*pyr*G_2 T>C)
**UT**	18Y0125	Milk powder	2006	8	1	**~5**	3	6	2	3	Similar to ST/152 with a difference in one nucleotide (Pas_gltA_5 T >C)
	18Y0143	Milk powder	2006	8	1	**~5**	3	6	2	3	
	18Y0180	Milk powder	2008	8	1	**~5**	3	6	2	3	
	18Y0190	Milk powder	2009	8	1	**~5**	3	6	2	3	

* Novel Allele, alleles found with less than 100% identity. (~n) means novel full-length allele similar to n. UT, untyped strains.

**Table 4:** Strain types (ST) of 89 *A. baumannii* strains isolated in Germany based on the Oxford scheme.

Oxford ST.	No. of Isolates	%	Origin of Isolates
**ST/613**	19	21.4	Milk powder
**ST/427**	10	11.2	Milk powder
**ST/1182**	1	1.1	Milk powder
**ST/1418**	2	2.3	Human
**ST/195**	1	1.1	Human
**ST/348**	1	1.1	Human
**ST/806**	1	1.1	Human
**ST/944**	1	1.1	Human
**ST/931**	1	1.1	DSM30007 (Human)
**ST/112**	1	1.1	DSM105126 (Human)
**ND**	51	57.4	41 Milk powder; 8 Human; 2 Animal
**Total**	89	100	

ST, sequence type; ND, not determined.

**Table 5:** Allelic profiles of the untyped STs found in the current study based on the Oxford scheme (*Acinetobacter baumannii*#1).

ST	ID	*glt*A	*gyr*B	*gdh*B	*rec*A	*cpn*60	*Gpi*	*rpo*D	Novel Allele *
**UT**	18Y0036	1	121	2	2	36	98	30	
**UT**	18Y0093	2	97	~73	1	1	68	~6	(Oxf_*gdh*B_73 T>G, T>C) (Oxf_*rpo*D_6 T>G) (Oxf_*gpi*_68 T>C)
	18Y0112	2	97	~73	1	1	68	~6	
**UT**	18Y0111	1	113	~189	2	1	253	4	(Oxf_*gdh*B_66 A>G)
**UT**	18Y0141	1	47	140	6	1	~264	43	Oxf_*gpi*_264 T>C, C>G, A>G, G>T, G>A, T>C, A>T, T>C, T>C)
**1182**	18Y0169	1	12	56	1	1	107	26	
**UT**	18Y0192	35	31	49	11	~1	54	~5	(Oxf_*cpn*60_1 C>A) (Oxf_*rpo*D_5 T>C)
**UT**	18Y0245	1	15	59	28	94	157	45	Oxf_*cpn*60 has uncertain hit, and ST is unclear
**UT**	18Y0092	~1	17	42	60	4	140	151	(Oxf_*glt*A_1 G>A)
**UT**	18Y0103	24	52	139	1	~44	99	50	(Oxf_*cpn*60_26 G>A)
	18Y0107	24	52	139	1	~44	99	50	
	18Y0237	24	52	139	1	~44	99	50	
	18Y0228	24	52	139	1	~44	99	50	(Oxf_*cpn*60_16 C>T)
**UT**	18Y0195	1	41	186	11	1	164	6	
**UT**	18Y0125	98	12	40	26	32	103	4	
	18Y0143	98	12	40	26	32	103	4	
	18Y0180	98	12	40	26	32	103	4	
	18Y0190	98	12	40	26	32	103	4	

* Novel Allele, alleles found with less than 100% identity and ST might indicate nearest ST. (~n) means novel full-length allele similar to n. UT, untyped ST.

## References

[1] Howard A., O’Donoghue M., Feeney A., Sleator R.D. (2012). *Acinetobacter baumannii:* An emerging opportunistic pathogen. Virulence.

[2] Wareth G., Brandt C., Sprague L.D., Neubauer H., Pletz M.W. (2020). Spatio-temporal distribution of *Acinetobacter baumannii* in Germany-A comprehensive systematic review of studies on resistance development in humans (2000–2018). Microorganisms.

[3] Ewers C., Klotz P., Leidner U., Stamm I., Prenger-Berninghoff E., Gottig S., Semmler T., Scheufen S. (2017). OXA-23 and ISAba1-OXA-66 class D beta-lactamases in *Acinetobacter baumannii* isolates from companion animals. Int. J. Antimicrob. Agents.

[4] Klotz P., Gottig S., Leidner U., Semmler T., Scheufen S., Ewers C. (2017). Carbapenem-resistance and pathogenicity of bovine *Acinetobacter* indicus-like isolates. PLoS ONE.

[5] Wareth G., Abdel-Glil M.Y., Schmoock G., Steinacker U., Kaspar H., Neubauer H., Sprague L.D. (2019). Draft genome sequence of an *Acinetobacter baumannii* isolate recovered from a horse with conjunctivitis in Germany. Microbiol. Resour. Announc..

[6] Cho G.S., Li B., Rostalsky A., Fiedler G., Rosch N., Igbinosa E., Kabisch J., Bockelmann W., Hammer P., Huys G. (2018). Diversity and antibiotic susceptibility of *Acinetobacter* strains from milk powder produced in Germany. Front. Microbiol..

[7] Wareth G., Linde J., Hammer P., Nguyen N.H., Nguyen T.N.M., Splettstoesser W.D., Makarewicz O., Neubauer H., Sprague L.D., Pletz M.W. (2020). Phenotypic and WGS-derived antimicrobial resistance profiles of clinical and non-clinical *Acinetobacter baumannii* isolates from Germany and Vietnam. Int. J. Antimicrob. Agents.

[8] Wareth G., Neubauer H., Sprague L.D. (2019). *Acinetobacter baumannii*—A neglected pathogen in veterinary and environmental health in Germany. Vet. Res. Commun..

[9] Nemec A., Krizova L., Maixnerova M., Sedo O., Brisse S., Higgins P.G. (2015). *Acinetobacter seifertii* sp. nov., a member of the *Acinetobacter calcoaceticus-Acinetobacter baumannii* complex isolated from human clinical specimens. Int. J. Syst. Evol. Microbiol..

[10] Cosgaya C., Mari-Almirall M., Van Assche A., Fernandez-Orth D., Mosqueda N., Telli M., Huys G., Higgins P.G., Seifert H., Lievens B. (2016). *Acinetobacter dijkshoorniae* sp. nov., a member of the *Acinetobacter calcoaceticus-Acinetobacter baumannii* complex mainly recovered from clinical samples in different countries. Int. J. Syst. Evol. Microbiol..

[11] Higgins P.G., Schneiders T., Hamprecht A., Seifert H. (2010). In Vivo selection of a missense mutation in *ade*R and conversion of the novel *bla*OXA-164 gene into *bla*OXA-58 in carbapenem-resistant *Acinetobacter baumannii* isolates from a hospitalized patient. Antimicrob. Agents Chemother..

[12] Jain C., Rodriguez R.L., Phillippy A.M., Konstantinidis K.T., Aluru S. (2018). High throughput ANI analysis of 90K prokaryotic genomes reveals clear species boundaries. Nat. Commun..

[13] Wood D.E., Salzberg S.L. (2014). Kraken: Ultrafast metagenomic sequence classification using exact alignments. Genome Biol..

[14] Bartual S.G., Seifert H., Hippler C., Luzon M.A., Wisplinghoff H., Rodriguez-Valera F. (2005). Development of a multilocus sequence typing scheme for characterization of clinical isolates of *Acinetobacter baumannii*. J. Clin. Microbiol..

[15] Diancourt L., Passet V., Nemec A., Dijkshoorn L., Brisse S. (2010). The population structure of *Acinetobacter baumannii*: Expanding multiresistant clones from an ancestral susceptible genetic pool. PLoS ONE.

[16] Gaiarsa S., Batisti Biffignandi G., Esposito E.P., Castelli M., Jolley K.A., Brisse S., Sassera D., Zarrilli R. (2019). Comparative analysis of the two *Acinetobacter baumannii* multilocus sequence typing (MLST) schemes. Front. Microbiol..

[17] Kenyon J.J., Hall R.M. (2013). Variation in the complex carbohydrate biosynthesis loci of *Acinetobacter baumannii* genomes. PLoS ONE.

[18] Murugaiyan J., Walther B., Stamm I., Abou-Elnaga Y., Brueggemann-Schwarze S., Vincze S., Wieler L.H., Lubke-Becker A., Semmler T., Roesler U. (2014). Species differentiation within the *Staphylococcus* intermedius group using a refined MALDI-TOF MS database. Clin. Microbiol. Infect. Off. Publ. Eur. Soc. Clin. Microbiol. Infect. Dis..

[19] Turton J.F., Woodford N., Glover J., Yarde S., Kaufmann M.E., Pitt T.L. (2006). Identification of *Acinetobacter baumannii* by detection of the *bla*OXA-51-like carbapenemase gene intrinsic to this species. J. Clin. Microbiol..

[20] Woodford N., Ellington M.J., Coelho J.M., Turton J.F., Ward M.E., Brown S., Amyes S.G., Livermore D.M. (2006). Multiplex PCR for genes encoding prevalent OXA carbapenemases in *Acinetobacter* spp.. Int. J. Antimicrob. Agents.

[21] Kobs V.C., Ferreira J.A., Bobrowicz T.A., Ferreira L.E., Deglmann R.C., Westphal G.A., França P.H. (2016). The role of the genetic elements *bla*OXA and IS Aba 1 in the *Acinetobacter calcoaceticus-Acinetobacter baumannii* complex in carbapenem resistance in the hospital setting. Rev. Soc. Bras. Med. Trop.

[22] Wood D.E., Lu J., Langmead B. (2019). Improved metagenomic analysis with Kraken 2. Genome Biol..

[23] Bankevich A., Nurk S., Antipov D., Gurevich A.A., Dvorkin M., Kulikov A.S., Lesin V.M., Nikolenko S.I., Pham S., Prjibelski A.D. (2012). SPAdes: A new genome assembly algorithm and its applications to single-cell sequencing. J. Comput. Biol. A J. Comput. Mol. Cell Biol..

[24] Gurevich A., Saveliev V., Vyahhi N., Tesler G. (2013). QUAST: Quality assessment tool for genome assemblies. Bioinformatics.

[25] Richter M., Rossello-Mora R. (2009). Shifting the genomic gold standard for the prokaryotic species definition. Proc. Natl. Acad. Sci. USA.

[26] Klotz P., Higgins P.G., Schaubmar A.R., Failing K., Leidner U., Seifert H., Scheufen S., Semmler T., Ewers C. (2019). Seasonal occurrence and carbapenem susceptibility of bovine *Acinetobacter baumannii* in Germany. Front. Microbiol..

[27] Vijayakumar S., Biswas I., Veeraraghavan B. (2019). Accurate identification of clinically important *Acinetobacter* spp.: An update. Future Sci. OA.

[28] Szabados F., Tix H., Anders A., Kaase M., Gatermann S.G., Geis G. (2012). Evaluation of species-specific score cutoff values of routinely isolated clinically relevant bacteria using a direct smear preparation for matrix-assisted laser desorption/ionization time-of-flight mass spectrometry-based bacterial identification. Eur. J. Clin. Microbiol. Infect. Dis. Off. Publ. Eur. Soc. Clin. Microbiol..

[29] Turton J.F., Ward M.E., Woodford N., Kaufmann M.E., Pike R., Livermore D.M., Pitt T.L. (2006). The role of ISAba1 in expression of OXA carbapenemase genes in *Acinetobacter baumannii*. FEMS Microbiol. Lett..

[30] Wendel A.F., Malecki M., Otchwemah R., Tellez-Castillo C.J., Sakka S.G., Mattner F. (2018). One-year molecular surveillance of carbapenem-susceptible *A. baumannii* on a German intensive care unit: Diversity or clonality. Antimicrob. Resist. Infect. Control.

[31] Hamidian M., Nigro S.J. (2019). Emergence, molecular mechanisms and global spread of carbapenem-resistant *Acinetobacter baumannii*. Microb. Genom..

[32] Molter G., Seifert H., Mandraka F., Kasper G., Weidmann B., Hornei B., Ohler M., Schwimmbeck P., Kroschel P., Higgins P.G. (2016). Outbreak of carbapenem-resistant *Acinetobacter baumannii* in the intensive care unit: A multi-level strategic management approach. J. Hosp. Infect..

[33] Eigenbrod T., Reuter S., Gross A., Kocer K., Gunther F., Zimmermann S., Heeg K., Mutters N.T., Nurjadi D. (2019). Molecular characterization of carbapenem-resistant *Acinetobacter baumannii* using WGS revealed missed transmission events in Germany from 2012–2015. J. Antimicrob. Chemother..

[34] Pfeifer Y., Hunfeld K.P., Borgmann S., Maneg D., Blobner W., Werner G., Higgins P.G. (2016). Carbapenem-resistant *Acinetobacter baumannii* ST78 with OXA-72 carbapenemase and ESBL gene *bla*CTX-M-115. J. Antimicrob. Chemother..

[35] Linz B., Mukhtar N., Shabbir M.Z., Rivera I., Ivanov Y.V., Tahir Z., Yaqub T., Harvill E.T. (2018). Virulent epidemic pneumonia in sheep caused by the human pathogen *Acinetobacter baumannii*. Front. Microbiol..

[36] Ewers C., Klotz P., Scheufen S., Leidner U., Göttig S., Semmler T. (2016). Genome sequence of OXA-23 producing *Acinetobacter baumannii* IHIT7853, a carbapenem-resistant strain from a cat belonging to international clone IC1. Gut Pathog..

[37] Frickmann H., Crusius S., Walter U., Podbielski A. (2010). Management eines Ausbruchs nosokomialer Pneumonien durch einen neuen multiresistenten *Acinetobacter baumannii*-Klon. Pneumologie.

[38] Savin M., Parcina M., Schmoger S., Kreyenschmidt J., Kasbohrer A., Hammerl J.A. (2019). Draft genome sequences of *Acinetobacter baumannii* isolates recovered from sewage water from a poultry slaughterhouse in Germany. Microbiol. Resour. Announc..

